# Architecture and functions of stomatal cell walls in eudicots and grasses

**DOI:** 10.1093/aob/mcae078

**Published:** 2024-05-17

**Authors:** Leila Jaafar, Charles T Anderson

**Affiliations:** Department of Biology and Molecular, Cellular and Integrative Bioscience Graduate Program, The Pennsylvania State University, University Park, PA 16802, USA; Department of Biology and Molecular, Cellular and Integrative Bioscience Graduate Program, The Pennsylvania State University, University Park, PA 16802, USA

**Keywords:** *Arabidopsis thaliana*, biomechanics, *Brachypodium distachyon*, cellulose, guard cells, hemicellulose, pectin, plant cell wall, stomatal complex

## Abstract

**Background:**

Like all plant cells, the guard cells of stomatal complexes are encased in cell walls that are composed of diverse, interacting networks of polysaccharide polymers. The properties of these cell walls underpin the dynamic deformations that occur in guard cells as they expand and contract to drive the opening and closing of the stomatal pore, the regulation of which is crucial for photosynthesis and water transport in plants.

**Scope:**

Our understanding of how cell wall mechanics are influenced by the nanoscale assembly of cell wall polymers in guard cell walls, how this architecture changes over stomatal development, maturation and ageing and how the cell walls of stomatal guard cells might be tuned to optimize stomatal responses to dynamic environmental stimuli is still in its infancy.

**Conclusion:**

In this review, we discuss advances in our ability to probe experimentally and to model the structure and dynamics of guard cell walls quantitatively across a range of plant species, highlighting new ideas and exciting opportunities for further research into these actively moving plant cells.

## INTRODUCTION

### Stomatal complex configuration and function

Stomatal complexes are flexible multicellular systems at the leaf surface that can open and close dynamically to regulate water transport and gas exchange for photosynthesis (Farquhar and Sharkey, 1982). Stomatal complexes possess two guard cells that separate in the middle to form the stomatal pore, where transpiration and gas exchange take place. Stomatal complexes can also help plants to regulate leaf temperature via evaporative cooling. In angiosperms, guard cells can swell and shrink reversibly to increase or decrease, respectively, the size of the stomatal pore in response to changes in environmental conditions such as light, temperature, humidity and CO_2_ levels. Stomata are thought to be sites for the emission of volatile signals that can travel across or between plants, and stomatal pore size can be influenced by pathogens and herbivores (Guimarães and Stotz, 2004; Melotto *et al.*, 2006; Allègre *et al.*, 2007).

The emergence of stomata in plants is one of the crucial landmarks in the adaptation of plants to terrestrial environments. Stomata are present in vascular plants and some bryophytes. Based on fossil, phylogenomic and physiological evidence across all land plants, it has been proposed that stomata arose initially as valves that allowed for the desiccation of the sporophyte, aiding in spore dispersal (Duckett *et al.*, 2009; Chater *et al.*, 2016; Renzaglia *et al.*, 2017; Kubásek *et al.*, 2021; McAdam *et al.*, 2021).

Different plant species display different configurations of stomatal complexes. Stomatal complexes in eudicots are composed of pairs of kidney-shaped guard cells, whereas stomatal complexes in grasses (Poaceae) contain two dumbbell-shaped guard cells that have bulbous ends connected with a central canal. Developing guard cells in many species, including grasses, recruit subsidiary cells that flank the guard cells and are hypothesized to aid in stomatal opening and/or closing (Gray *et al.*, 2020). For example, although kidney-shaped guard cells of *Arabidopsis thaliana* (Arabidopsis) lack morphologically distinct subsidiary cells, kidney-shaped guard cells of *Kalanchoe laxiflora* recruit several subsidiary cells that surround the guard cells on all sides (Nunes *et al.*, 2020). Myriad other arrangements of guard cells and subsidiary cells exist (Nunes *et al.*, 2020); for example, dumbbell-shaped guard cells in grasses recruit two flanking subsidiary cells that can be rectangular, such as those seen in *Brachypodium distachyon*, or dome-shaped, such as those seen in rice (*Oryza sativa*). This means that different cellular configurations in the stomatal complexes of different species achieve the same overall goal, which is to regulate pore size effectively in response to environmental and intrinsic cues, through different cellular mechanisms. Angiosperm stomatal opening is an active process, whereby guard cells have to overcome the mechanical advantage exerted on them by neighbouring epidermal cells, whereas pore opening in fern and lycophyte stomata is a passive process that depends on the water content of the leaf, which might be attributable to the lack of mechanical advantage exerted on these guard cells by neighbouring epidermal cells (Pichaco *et al.*, 2024), which vary in their arrangement in these species in comparison to the anomocytic or graminoid arrangements of epidermal cells in *A. thaliana* or *B. distachyon* complexes, respectively (Nunes *et al.*, 2020).

**Fig. 1.**
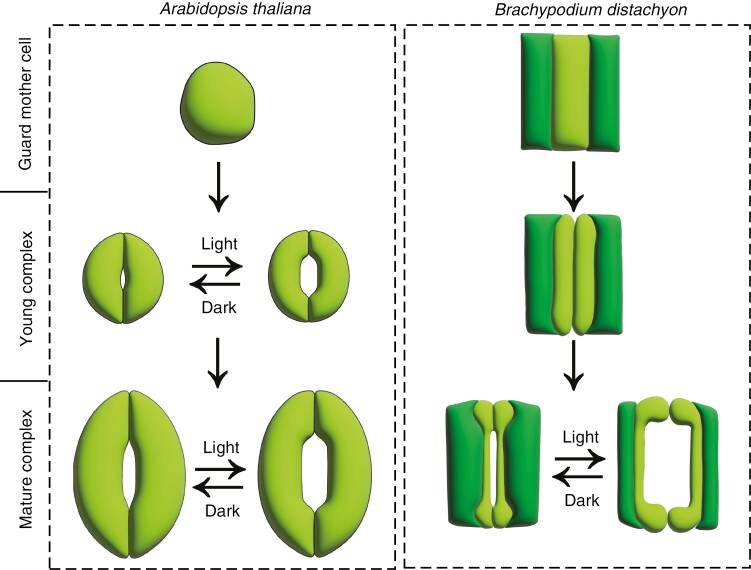
Differentiation and maturation of eudicot and grass stomatal complexes. In eudicots, e.g. *Arabidopsis thaliana*, a guard mother cell (left panel) divides symmetrically to give rise to two young kidney-shaped guard cells that separate in the middle to form the stomatal pore. Young stomatal complexes then grow and elongate to form mature complexes. Young and mature stomatal complexes can respond to opening and closing cues such as light and dark, respectively. In grasses, e.g. *Brachypodium distachyon*, the rectangular guard mother cell is flanked by two subsidiary cells (right panel). The guard mother cell divides symmetrically to give rise to two young guard cells and form a young stomatal complex. After pore formation and maturation via elongation of guard and subsidiary cells, the mature stomatal complex becomes responsive to environmental stimuli such as light and dark conditions.

**Fig. 2.**
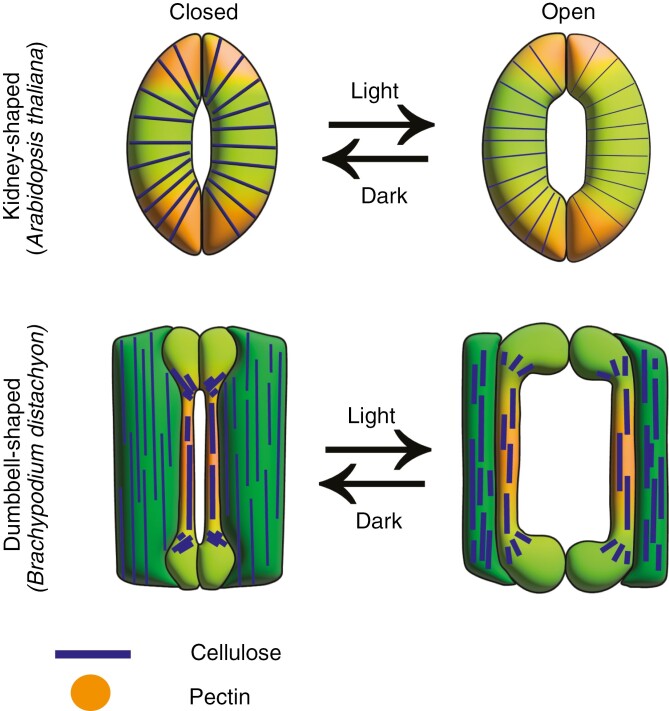
Models of the dynamics of cellulose and pectin organization during stomatal function in kidney-shaped and dumbbell-shaped guard cells. In kidney-shaped guard cells, such as those of *Arabidopsis thaliana*, cellulose wraps around the circumference of the cell. In response to pore-closing stimuli (e.g. dark), cellulose forms bundles. Conversely, in response to an opening stimulus (e.g. light), cellulose bundles separate to allow for the guard cell elongation. The poles of kidney-shaped guard cells are rich in low-methyl-esterified pectin that provides stiffness to pin down and constrain the overall elongation of the complex, leading to the lateral bending of the guard cells and the opening of the pore. Stomatal complexes of grasses, such as *Brachypodium distachyon*, contain dumbbell-shaped guard cells that are flanked by two subsidiary cells. In opening conditions, guard cells expand asymmetrically at the ventral wall, leading to the bulbous end bulging and opening the pore (Gkolemis *et al.*, 2023). We hypothesize that, in response to opening conditions (e.g. light), in which subsidiary cells compress to accommodate the guard cells, longitudinally arranged cellulose in subsidiary cells coalesces to form bundles; conversely, in response to closing conditions (e.g. dark), cellulose bundles separate to allow for subsidiary cell expansion and closing of the pore. We also hypothesize that longitudinally arranged cellulose in the central canal of the dumbbell-shaped guard cells, which is rich in low-methyl-esterified pectin, might not undergo reorganization owing to the stiffness of this region.

This leaves us wondering why all species do not have the same guard cell shapes and cellular configurations in their stomatal complexes. Why has this diversity arisen evolutionarily? The functions of stomatal complexes are tightly related to environmental conditions that can vary widely across different biomes. Thus, we speculate that different pore sizes and speeds of stomatal response might allow species to adapt to differing combinations and patterns of environmental stimuli. Comparing the functions of kidney-shaped and dumbbell-shaped guard cells can provide insight into the importance of variation in stomatal architecture (Fig. 1): in *A. thaliana*, a eudicot that has kidney-shaped guard cells, turgor pressure increases in response to opening stimuli, leading to guard cell inflation and elongation. Given that sister guard cells are connected at their junctions by stiff poles that prevent the overall lengthening of the stomatal complex (Carter *et al.*, 2017), elongating guard cells will bend to separate laterally and open the pore. The opposite happens in conditions that stimulate stomatal closure (e.g. darkness), whereby turgor pressure decreases, and the guard cells deflate and shrink to close the pore and limit water loss (Fig. 2) (Rui and Anderson, 2016; Carter *et al.*, 2017). In contrast, in grass (graminoid) stomata with dumbbell-shaped guard cells flanked by two subsidiary cells, different models have been proposed to explain the biomechanics of such complexes. The first is the see-saw model, in which the main driver of the stomatal function is an inversely proportional change in turgor pressure between guard and subsidiary cells (Franks and Farquhar, 2007). In this model, in opening conditions the turgor pressure increases in the guard cells and decreases in the subsidiary cells, allowing the guard cells to push against them to open the pore. This cellular interplay results in a pore area that is larger in proportion to the size of the stomatal complex than in stomatal complexes with kidney-shaped guard cells. In closing conditions, the turgor pressure in the guard cells decreases while that in the subsidiary cells increases to push the guard cells together and close the pore (Franks and Farquhar, 2007). The second model is the spring model for grass stomatal function states that although the inversely proportional change in turgor pressure between guard and subsidiary cells is important to achieve a more efficient stomatal function, subsidiary cells are not essential to achieve stomatal dynamics. This is based on observations of a mutant of *B. distachyon* that lacks subsidiary cells (*Bdmute*), in which guard cells are able to open and close the stomatal pore without the assistance of subsidiary cells, albeit to a smaller degree than in wild-type complexes (Raissig *et al.*, 2017; Durney *et al.*, 2023). MUTE is a transcription factor important for the establishment of subsidiary cells in grasses such as *B. distachyon*, *Zea mays* and *O. sativa* (Raissig *et al.*, 2017; Wang *et al.*, 2019; Wu *et al.*, 2019; Zhang *et al.*, 2022). Single defective or missing subsidiary cells in stomatal complexes of polarity mutants in maize (*Pan2*) curved the guard cells towards the remaining subsidiary cell (Liu *et al.*, 2024). This means that subsidiary cells are crucial to hold the central canal of the guard cells and maintain its geometry, which was shown to be important for stomatal function (Durney *et al.*, 2023).

In either type of stomatal complex, guard cells should be highly flexible to withstand the constant deformations that accompany opening and closing of the stomatal pore in response to environmental stimuli. This deformability is hypothesized to be achieved by a flexible cell wall that maintains structural integrity in the face of the large amounts of turgor pressure that exist in many plant species (Franks and Farquhar, 2007), while allowing for changes in guard cell shape.

### Cell wall assembly and mechanics in stomatal complexes

The primary cell wall is a dynamic structure that maintains cellular integrity by forming a physical barrier between the cell and the external environment, resisting turgor pressure, enabling cell expansion and allowing for cell division and differentiation. The cell wall is made up of networks of polysaccharides and glycoproteins in addition to non-structural proteins and water (Anderson and Kieber, 2020). Polysaccharide polymers in the plant cell wall include pectins, hemicelluloses and cellulose.

Pectins are a class of biopolymers that are synthesized in the Golgi apparatus and secreted into the wall. Pectins are negatively charged, which allows them to be highly hydrated and/or crosslinked with ions to form gel structures (Du *et al.*, 2022). Pectins include three main domains: homogalacturonan, a linear chain of galacturonic acid residues; rhamnogalacturonan-I, which contains an alternating backbone of rhamnose and galacturonic acid residues decorated with arabinan and galactan side chains; and rhamnogalacturonan-II, which has a galacturonic acid backbone elaborated with complex side chains (Anderson, 2019). Pectin methyl-esterases remove ester groups from the extensively methyl-esterified homogalacturonan that is secreted into the wall. Specific patterns of de-methyl-esterification create a range of substrates for subsequent modifications. Homogalacturonans with randomly de-methyl-esterified groups are substrates for pectin-degrading enzymes such as polygalacturonases and pectate lyases, the activity of which can facilitate cell expansion in growing tissues (Xiao *et al.*, 2014). Homogalacturonans with continuous stretches of de-methyl-esterified galacturonic acids can be substrates for calcium cross-linking that creates a stiff eggbox structure potentially constraining cell expansion (Ha *et al.*, 1997; Braccini and Pérez, 2001; Vincken *et al.*, 2003; Du *et al.*, 2022; Temple *et al.*, 2022).

Hemicelluloses are also synthesized in the Golgi apparatus and secreted into the wall via exocytosis. These polymers are modified in the wall after secretion by endohydrolases that degrade hemicelluloses and endotransglycosylases that can cleave and then link different hemicellulose molecules to form new polymers and networks (Fry *et al.*, 1992; Nishitani and Tominaga, 1992; Thompson and Fry, 2001; Fry, 2004). In at least some cases, hemicelluloses can bind to cellulose microfibrils at ‘biomechanical hotspots’ that hinder cell wall extensibility and are thought to be the sites of action for expansins, non-enzymatic proteins that induce wall creep (Cosgrove, 2022). Interestingly, experimental evidence indicates that expansins can accelerate stomatal opening in Arabidopsis (Wei *et al.*, 2011; Zhang *et al.*, 2011), implying that rearrangement of the cellulose–hemicellulose network might constitute an important process during guard cell deformation. Defects in stomatal opening and closure in an Arabidopsis mutant lacking one type of hemicellulose, xyloglucan (Rui and Anderson, 2016), support this idea.

Cellulose is an important load-bearing polymer in the cell wall (Zhang *et al.*, 2017, 2021). Cellulose is synthesized at the plasma membrane by enzymatic complexes and is deposited as β-1,4-glucan chains that coalesce to form microfibrils (Chan and Coen, 2020). The pattern of cellulose deposition can be dictated either by cortical microtubules or by patterning in the pre-deposited cell wall (Chan and Coen, 2020). This microtubule-driven cellulose patterning is evident in Arabidopsis guard cells that have circumferentially arranged cellulose microfibrils and microtubules that radiate from the centre of the complex (Lucas *et al.*, 2006; Fujita and Wasteneys, 2014; Rui and Anderson, 2016; Jaafar *et al.*, 2024).

Cell growth in plants is hypothesized to involve a transient increase in cytoplasmic turgor pressure that imposes an internal force on the cell wall, which then yields, leading to irreversible cell expansion. The Lockhart equation models plant cell growth as a viscoelastic behaviour that depends on turgor pressure exceeding a given yield threshold, and cell extensibility (Lockhart, 1965). However, this model does not connect cell wall structure and dynamics to cell growth mechanics. Several models have modified the Lockhart model to account for cell wall structure, wall loosening and hardening and the anisotropic mechanical properties of the wall (Dumais *et al.*, 2006; Dyson and Jensen, 2010; Chakraborty *et al.*, 2021). During wall extension imposed by an external force, cellulose microfibrils have been observed to straighten, curve, slide against one another or reorient (Zhang *et al.*, 2017). Modelling has indicated that these behaviours of cellulose microfibrils depend on the direction of expansion (Zhang *et al.*, 2021). Computational simulations of the opening of kidney-shaped guard cells have predicted that water influx rather than turgor pressure is a primary driver of stomatal opening (Yi *et al.*, 2022). These simulations showed that, in the case of an opening stimulus, water influx leads to an initial transient increase in turgor pressure. The compliance of the cell wall and the continuous influx of water as the cell expands lead to longitudinal expansion of the guard cells, which are pinned down at their poles owing to stiff walls and balancing forces imposed by junctional pavement cells (Carter *et al.*, 2017; Davaasuren *et al.*, 2022), resulting in lateral cell bending and pore opening (Yi *et al.*, 2022).

In helping plants to respond to rapidly changing environmental conditions, e.g. clouds sporadically blocking sunlight, kidney-shaped guard cells and grass subsidiary cells display reversible deformation in the form of frequent expansion and compression that are contingent on external stimuli. Although it is currently unclear how cell wall arrangement in grass subsidiary cells affects their flexibility, the cell walls of kidney-shaped stomata of Arabidopsis have been studied extensively (Fujita and Wasteneys, 2014; Amsbury *et al.*, 2016; Rui and Anderson, 2016; Carter *et al.*, 2017; Rui *et al.*, 2019; Keynia *et al.*, 2023). In response to opening conditions, cellulose microfibril bundles in kidney-shaped guard cells reorganize and separate, whereas in closing conditions the cellulose microfibrils bundle (Fig. 2) (Rui and Anderson, 2016).

Below, we review the composition and organization of cell walls in stomatal complexes containing kidney-shaped and dumbbell-shaped guard cells from a developmental perspective, starting from stomatal differentiation through pore formation and, finally, stomatal maturation and function (Fig. 1), highlighting recent advances and pointing out knowledge gaps and new hypotheses regarding the relationships between cell wall architecture, wall and cellular biomechanics and stomatal dynamics.

## CELL WALL DYNAMICS DURING STOMATAL DIFFERENTIATION

A cascade of transcription factors drives kidney-shaped stomatal differentiation in the leaf epidermis in *A. thaliana*. Under the action of SPEECHLESS, protodermal cells divide to give rise to meristemoid cells that divide asymmetrically to create a guard mother cell under the action of a second transcription factor, MUTE. A third transcription factor, FAMA, then induces the symmetrical division of the guard mother cell, characterized by cell wall thickening (Zhao and Sack, 1999), into two guard cells (Pillitteri *et al.*, 2007; Hachez *et al.*, 2011; Guo *et al.*, 2021). During cytokinesis, the cell plate is formed by the delivery of cytokinetic vesicles to the division site (Samuels *et al.*, 1995; Seguí-Simarro *et al.*, 2004). Callose is then deposited transiently (Samuels *et al.*, 1995). The cell plate then matures by smoothening and expanding. This is followed by hypothesized stiffening of the cell plate through cellulose deposition (Miart *et al.*, 2014) in addition to the delivery of other polysaccharides. Finally, the mature cell plate fuses with the parent cell membrane and wall, marking the completion of cytokinesis (Fig. 1) (Samuels *et al.*, 1995; Seguí-Simarro *et al.*, 2004). Little is known about pectin and hemicellulose organization in the walls of sister guard cells immediately after cytokinesis and before stomatal pore formation. However, cellulose microfibril orientation in newly divided guard cells has been analysed (Rui and Anderson, 2016). Cellulose synthase tracks align with the radially arranged microtubules, which reflect the organization of cellulose microfibrils in these nascent complexes (Rui and Anderson, 2016), implying that cellulose begins to be wrapped circumferentially around guard cells soon after division.

In the model grass species, *B. distachyon*, stomatal complex formation is initiated by SPEECHLESS, which dictates cell fate and gives rise via asymmetric cell division to a guard mother cell. Subsequently, the guard mother cell expresses the transcription factor MUTE, which leaks into neighbouring epidermal cells and induces their asymmetrical division to generate the two subsidiary cells that flank the guard cells (Raissig *et al.*, 2017). Finally, the guard mother cell divides symmetrically to create two dumbbell-shaped guard cells (Rudall *et al.*, 2013), completing the formation of the stomatal complex (Fig. 1).

Although grasses possess type II cell walls that have lower pectin and higher xylan content (Smith and Harris, 1999; Vogel, 2008), immunolabelling experiments targeting the cell walls of differentiating grass stomata in *Z. mays* have revealed uneven distributions of pectic polysaccharides (Giannoutsou *et al.*, 2016). Immunolabelling results indicated that highly methyl-esterified pectin is present in the wall that separates the guard and subsidiary cells in young maize stomata before pore formation. Pectin with a low degree of methyl-esterification detected by JIM5 antibodies is mainly detected in the anticlinal wall and the middle of the periclinal walls of guard cells in young complexes. Calcium cross-linkable pectin is localized at the transverse walls of young guard cells that are shared with the subsidiary cells in young stomatal complexes (Giannoutsou *et al.*, 2016). Similar to the kidney-shaped stomata of Arabidopsis, little is known about hemicellulose and cellulose configurations and functions in the grass stomatal lineage.

## STOMATAL PORE FORMATION GIVES RISE TO YOUNG STOMATAL COMPLEXES

For proper pore formation, sister guard cells should separate only at the centre of their common walls. Time-lapse imaging of developing stomatal complexes in Arabidopsis cotyledons coupled with treatments with pectin-degrading enzymes and osmotic manipulations revealed that pore formation in Arabidopsis is achieved by a combination of pectin degradation and guard cell pressurization (Rui *et al.*, 2019). Interestingly, treatment with other cell wall-degrading enzymes, such as cellulases or hemicellulases, did not affect the timing of stomatal pore formation. These and other results gave rise to a model in which the deposition of methyl-esterified pectin and pectin-modifying enzymes, such as pectin methylesterases and polygalacturonases, into the cell wall initiates the localized separation of the kidney-shaped guard cells in Arabidopsis. Pore initiation is followed by pore enlargement that requires continued pectin degradation and sustained turgor pressure (Rui *et al.*, 2019). Little is known about how pores form in grass stomatal complexes, but in all plant species a young stomatal complex is formed upon the completion of pore formation.

In Arabidopsis, microscopic and spectroscopic evidence showed that young stomatal pores are covered by a waxy cuticle that might hinder guard cell movement to regulate pore size and also influence gas exchange (Hunt *et al.*, 2017). However, a stomatal dynamics assay comparing the function of young guard cells with mature ones illustrated the ability of young stomatal complexes to respond to light and dark conditions by opening and closing (Fig. 1) (Jaafar *et al.*, 2024). Cell wall mechanics in young Arabidopsis guard cells have been investigated. Lateral nanoindentation, which measures cell stiffness in the longitudinal, circumferential and radial directions (Keynia *et al.*, 2023), was used to generate three-dimensional stiffness profiles of young guard cells (Jaafar *et al.*, 2024). Finite element analysis of stiffness measurements, cell geometry and boundary properties predicts that the walls of young guard cells are mechanically isotropic, with the longitudinal wall modulus (E1) being similar to the circumferential wall modulus (E2) (Jaafar *et al.*, 2024). This implies that the degree of deformation of the cell wall would be expected to be the same along the longitudinal and the circumferential directions in response to an elevation in turgor pressure (Jaafar *et al.*, 2024); however, the elongated geometry of the guard cells might still favour elongation over widening of these cells during pressurization. Careful measurements of the geometry of individual guard cells in young stomatal complexes before and after opening will be required to test this idea.

Measurements of cell wall stiffness at the outer circumference of young stomatal complexes collected by atomic force microscopy have revealed a ‘polar stiffening’ characteristic that continues into mature stages of stomatal development (Carter *et al.*, 2017). Likewise, labelling with chitosan oligosaccharide (COS^488^) or JIM5 antibodies, both of which recognize low methyl-esterified pectin that can be crosslinked by calcium and potentially stiffen the wall, has revealed a high signal at the poles of *A. thaliana* and *Vigna seninses* stomatal complexes (Mravec *et al.*, 2014; Carter *et al.*, 2017; Giannoutsou *et al.*, 2020). The polar stiffening of stomatal complexes mediated by pectin modification is hypothesized to create a mechanical constraint on the length of the complex that leads to efficient stomatal opening (Fig. 2) (Carter *et al.*, 2017). However, direct experimental analysis of how the abundance or arrangement of hemicelluloses contributes mechanistically to the function or morphogenesis of young stomatal complexes, in either eudicots or grasses, is required to test the above hypotheses.

One question that arises from the above results is how the nanoscale architecture of the cell walls in young stomatal complexes gives rise to their isotropic mechanical properties and anisotropic cellular deformation. Imaging of green fluorescent protein-tagged cellulose synthase complexes and microtubules in young stomatal complexes implies that cellulose is deposited circumferentially around young guard cells in Arabidopsis, whereas field-emission scanning electron microscopy imaging of the innermost cell wall layer of young guard cells shows a random, isotropic organization of wall material (Fujita and Wasteneys, 2014). However, Pontamine Fast Scarlet 4B (S4B) staining shows that cellulose microfibrils are wrapped around the circumference of the young guard cells in *A. thaliana* (Jaafar *et al.*, 2024). These apparently contradictory observations might arise from the sample preparation protocol for field-emission scanning electron microscopy samples in the study by Fujita and Wasteneys (2014), in which wall components other than cellulose (such as pectins) were not removed from the sample, or might be attributable to the ability of S4B staining and confocal microscopy to image the entire thickness of the wall, in comparison to field-emission scanning electron microscopy, which images only the inner-most surface of the wall. Thus, the isotropic mechanical characteristics of young guard cells might be attributable to either fewer (but still anisotropically arranged) layers of cellulose in young guard cells, differences in the abundance, degree of crosslinking or organization of pectins, or other factors.

## STOMATAL MATURATION, MATURE FUNCTION AND AGEING

Stomatal maturation involves the growth of the stomatal complex to reach its final geometric dimensions and functional capacity. In Arabidopsis, stomatal complexes mature via the elongation of both the guard cells and the pore along a set of defined geometric milestones (Jaafar *et al.*, 2024). Stomatal complexes follow a similar trend of maturation in grasses, whereby the initially rectangular guard cells elongate and thin in their middle regions to form dumbbell-shaped cells, and the subsidiary cells also elongate (Hepworth *et al.*, 2018).

**Fig. 3.**
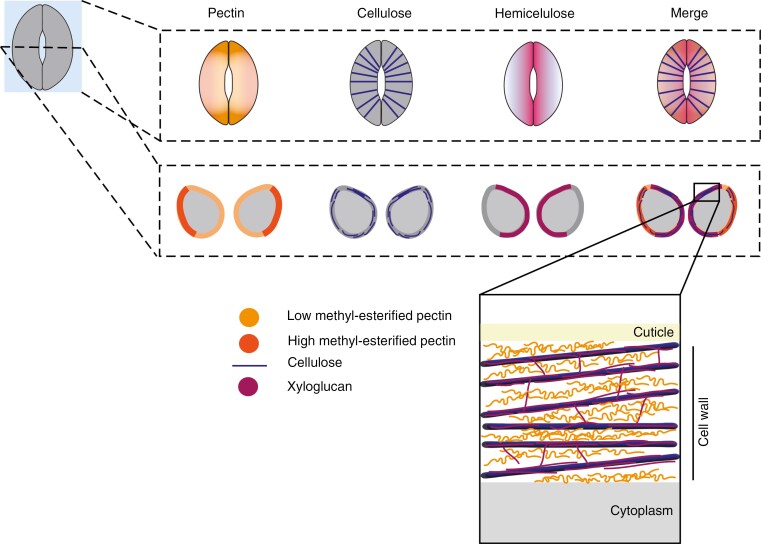
Cell wall architecture in kidney-shaped guard cells of *Arabidopsis thaliana*. In coronal (top row) and transverse (middle row) cross-sections of kidney-shaped guard cells of *A. thaliana*, low-methyl-esterified pectin is present at the poles and the ventral walls, whereas high-methyl-esterified pectin is evenly distributed at the dorsal wall. Cellulose is circumferentially arranged, and the hemicellulose, xyloglucan, occupies the ventral wall. A close-up model of the cell wall (lower panel) shows crosslinking of circumferentially arranged cellulose by xyloglucan and its spacing via pectin.

Pectin immunolabelling in sections of mature stomatal complexes from Arabidopsis leaves shows that guard cells are rich in unesterified homogalacturonans (Amsbury *et al.*, 2016). Stomatal complexes in an Arabidopsis mutant lacking a pectin methylesterase (*pme6*) were unable to respond normally to different experimental conditions, such as varying carbon dioxide levels, drought stress and temperature (Amsbury *et al.*, 2016), indicating that pectin de-methyl-esterification is a crucial step in the maturation of the guard cell wall. Additional pectin methylesterases have also been found to influence stomatal function (Huang *et al.*, 2017; Yang *et al.*, 2020; Wu *et al.*, 2022). In concordance with this, similar to young stomata, mature guard cells in Arabidopsis display stiff junctions that are also rich in low-methyl-esterified homogalacturonans (Fig. 3) (Carter *et al.*, 2017). In *Funaria hygrometrica*, a moss with doughnut-shaped stomatal complexes containing single, binucleate guard cells, pectin content decreases through a maturation process characterized by thickening cell walls that diminish in flexibility and movement (Merced and Renzaglia, 2014). In contrast, guard cell walls in sugar beet (*Beta vulgaris* L.) stomatal complexes are rich in methyl-esterified pectin (Majewska-Sawka *et al.*, 2002). Pectin modification by polygalacturonases and pectate lyases has also been implicated in the maturation and function of Arabidopsis stomatal complexes (Rui *et al.*, 2017; Chen *et al.*, 2021; Keynia *et al.*, 2023). Together, these data demonstrate the importance of pectins in the functions of kidney-shaped guard cells.

In addition to shape, during the maturation of the Arabidopsis stomatal complex the guard cell wall acquires mechanical anisotropy, whereby the circumferential Young’s modulus increases while the longitudinal modulus remains unchanged (Jaafar *et al.*, 2024). This mechanical anisotropy might arise from continued deposition of circumferentially arranged cellulose microfibrils during stomatal maturation (Fujita and Wasteneys, 2014; Rui and Anderson, 2016; Jaafar *et al.*, 2024). However, cellulose is also circumferentially organized in young guard cells (Jaafar *et al.*, 2024), which hints at potential roles for other wall polymers in establishing mechanical anisotropy in the wall. Mechanical analyses of cell wall mutants have recently been used to help determine how other wall polymers might establish mechanical anisotropy in the walls of mature kidney-shaped guard cells. In addition, functional analyses of the same mutants can test the implications of mechanical aberrations on stomatal function. For example, stomata of a cellulose synthase mutant (*cesa3*^*je5*^) that possesses lower levels of cellulose can respond to changes in light by opening or closing their pores (Jaafar *et al.*, 2024), but the size of the stomatal pore differs from wild-type controls (Rui and Anderson, 2016), and a smaller number of mature stomata are evident than in the wild type (Jaafar *et al.*, 2024). These data imply that cellulose microfibrils are required for normal stomatal maturation and function in Arabidopsis. Additional experiments with wall-modifying enzymes, for example those that degrade the arabinans and galactans that decorate rhamnogalacturonan-I and arabinogalactan proteins (Jones *et al.*, 2003, 2005), could also be informative in dissecting the mechanical influences of different wall polymers on stomatal maturation and dynamics.

Immunolabelling of the most abundant hemicellulose in eudicot primary walls, xyloglucan, using the LM25 antibody shows xyloglucan labelling around all regions of the wall except the dorsal wall contacting neighbouring cells in transverse cross-sections of Arabidopsis guard cells (Fig. 3) (Amsbury *et al.*, 2016). In addition, the xyloglucan-deficient mutant *xxt1 xxt2* displays smaller stomatal complexes and smaller apertures in response to opening and closing stimuli compared with wild-type controls (Rui and Anderson, 2016). These results suggest the importance of xyloglucan in maintaining the growth potential and flexibility of kidney-shaped guard cells in Arabidopsis. It would be interesting to test stomatal morphogenesis and function in the *xxt1 xxt2 csla2* mutant, which is dysfunctional for biosynthetic genes for both xyloglucan and mannan and has been reported to have more severe growth defects than *xxt1 xxt2* mutants (Yu *et al.*, 2022).

Upon opening and closing, the cell wall must undergo remodelling to expand and contract and thus coordinate with an increase or decrease in turgor pressure, respectively. Remodelling the guard cell wall can occur through either polysaccharide degradation or polymer reorganization. Cell wall degradation and deposition are lengthy, energy-consuming processes that might not be adaptive in the context of rapid, repeated stomatal responses to stimuli, which can occur in a matter of minutes. Evidence supporting polymer reorganization during stomatal dynamics is observed in the reorganization of cellulose, which appears to bundle in closed states and become more widely spaced in open states of Arabidopsis guard cells (Rui and Anderson, 2016). This cellulose reorganization is less evident in a cellulose-deficient mutant *cesa3*^*je5*^ (Desprez *et al.*, 2007) and the xyloglucan synthesis mutant *xxt1xxt2* (Cavalier *et al.*, 2008), further supporting cell wall reorganization rather than degradation during stomatal dynamics and highlighting the importance of cellulose and hemicellulose in the function of kidney-shaped guard cells (Rui and Anderson, 2016). We hypothesize that reorganization of cell wall polymers extends to pectins, which might constitute a spacing buffer between circumferentially arranged cellulose microfibrils; this hypothesis could be tested by imaging cellulose organization in the guard cells of pectin-deficient mutants. However, it is also possible that polymer degradation is functionally important for stomatal opening and closing, both during stomatal morphogenesis and during the function of mature stomata, with the corollary that some polymer degradation products, e.g. oligogalacturonides, act as signalling molecules that relay the status of the guard cell wall to the protoplast.

In grass stomata, guard cells that start as rectangular after the division of the guard mother cell elongate and gain their characteristic dumbbell shape as they mature (Raissig *et al.*, 2017). Subsidiary cells elongate along with the guard cells to achieve the mature stomatal configuration (Galatis, 1980; Galatis and Apostolakos, 2004). Propidium iodide, which is positively charged, binds to negatively charged carboxyl groups on de-methyl-esterified galacturonic acid residues in pectins (Rounds *et al.*, 2011), and propidium iodide staining of grass stomata shows the highest signal in the central canal of the guard cells but not the subsidiary cells (Raissig *et al.*, 2017; Durney *et al.*, 2023). De-methyl-esterified pectin, which can be Ca^2+^-crosslinked, in the central canal of the guard cells might stiffen the cell wall in this region, which has been proposed to be important for function (Braccini and Pérez, 2001; Vincken *et al.*, 2003; Durney *et al.*, 2023). The low signal of propidium iodide in the subsidiary cells (Hepworth *et al.*, 2018) might be attributed to several possibilities: a higher degree of pectin methyl-esterification, thinner walls or low pectin content in these cells. The first two possibilities might explain the high flexibility of subsidiary cells, which compress to open the grass pore and expand to close it.

Polarized light microscopy and S4B staining of grass stomata of wheat, sorghum and barley show longitudinal arrangements of cellulose in the central canal of the dumbbell-shaped cells that radiate at the hinges that connect the central canal to the bulbous ends. At the bulbous ends, cellulose displays a parallel, longitudinal pattern (Shtein *et al.*, 2017; Durney *et al.*, 2023). In the same studies, it is claimed that the subsidiary cells also show a longitudinal arrangement of cellulose microfibrils (Shtein *et al.*, 2017; Durney *et al.*, 2023). However, the subsidiary cells are dark under polarized light and after staining with S4B, which might confound the interpretation of cellulose patterning in these cells. The differential cellulose signals between guard cells and subsidiary cells in imaging data might be attributable to differences in wall thickness, cellulose abundance or cell positioning in the tissue, where cells lie at different heights in the epidermis. In maize, the xyloglucan was found to be distributed along the central canal and bulbous regions of the guard cells (Gkolemis *et al.*, 2023). Little is known about the properties or function of hemicellulose in grass stomata, either during stomatal maturation or in mature stomatal complexes.

It is not known whether stomatal complexes retain their functionality throughout the lifetime of the plant epidermis in which they are embedded (Kane *et al.*, 2020). Comparing the pore size of young and mature Arabidopsis stomata, recent results have indicated that mature stomatal pores in seedlings grown under continuous light remain more open than the pores of young complexes in closing and opening conditions (Chen, 2021). This was also the case after killing the guard cells with a targeted Micropoint ablation laser, where the pore did not close completely in mature stomatal complexes. Further observations of stomatal dynamics indicated that some mature stomatal complexes were locked in open states and could not respond to dark conditions by closing their pores (Chen, 2021). This behaviour was attributed to the concept of stomatal ageing, whereby it is hypothesized that the cell wall becomes stiff as the complex matures and thus stops yielding to changes in turgor pressure or water influx (Chen, 2021). In line with this idea, reductions in stomatal conductance have been detected in older leaves of several plant species (Reich, 1984; Jordan and Brodribb, 2007). Whether stomatal ageing occurs consistently across plant species and/or arises from senescence of the leaf that results in less hydrated, stiffer cell walls or owing to programmed ageing processes in the guard cells *per se* is not well understood. Future work to engineer guard cells to prevent ageing and prolong stomatal function might enhance carbon uptake and plant productivity.

## CONCLUSIONS AND FUTURE RESEARCH

In summary, the functional connections between the nanoscale architecture of the guard cell wall, cell wall mechanics, guard cell biomechanics and stomatal function are beginning to emerge through a synergistic combination of genetic, cell biological, physiological, mechanical measurement and modelling approaches. To gain a full understanding of how cell wall structure and mechanics change over the development, maturation and ageing of stomatal complexes, it will be important to apply these approaches at precise stages along these time lines across a range of plant species. In particular, analyses of wall structure and mechanics in the context of grass stomatal complexes, which show more rapid responses to environmental stimuli and have been hypothesized to be a crucial adaptation for the colonization of semi-arid biomes by grasses (Franks and Farquhar, 2007; Lawson and Vialet-Chabrand, 2019), would help to reveal the mechanisms underlying the enhanced function of these stomatal complexes. Future studies could examine the mechanism of pore formation in grass stomata using treatments with cell wall-degrading enzymes and genetic approaches. It would also be interesting to unravel and compare the molecular pathways behind stomatal maturation and ageing in eudicots and grasses using genetic manipulation, physiological and imaging tools. In addition, investigating the organization of hemicellulose and pectin in grass stomatal complexes is crucial to understanding the high degree of flexibility of the subsidiary cells and the dynamics of the biomechanical interaction between subsidiary and guard cells in these complexes. This could be achieved by integrating enzymatic, immunolabelling, imaging, biomechanical measurement and computational modelling approaches. Ultimately, this knowledge could be useful for engineering stomatal kinetics and responses to improve water-use efficiency (Lawson and Blatt, 2014; Papanatsiou *et al.*, 2019) in food and bioenergy crops, allowing for better plant productivity in the increasingly challenging environmental conditions that are accompanying climate change. Answering these questions will get us closer to engineering stomata with higher performance, hence better crop plants.
